# A momentary biomarker for depressive mood

**DOI:** 10.1186/s40203-016-0017-6

**Published:** 2016-03-16

**Authors:** Jinhyuk Kim, Toru Nakamura, Yoshiharu Yamamoto

**Affiliations:** Graduate School of Education, The University of Tokyo, 7-3-1 Hongo, Bunkyo-ku, Tokyo 113-0033 Japan; Department of Psychosomatic Research, National Institute of Mental Health, National Center of Neurology and Psychiatry, 4-1-1 Ogawa-Higashi, Kodaira, Tokyo 187-8553 Japan

**Keywords:** Physical activity, Depressive mood, Major depressive disorders, Ecological momentary assessment, Psychobehavioral biomarker

## Abstract

Many biomarkers from genetic, neuroimaging, and biological/biochemical measures have been recently developed in order to make a shift toward the objective evaluation of psychiatric disorders. However, they have so far been less successful in capturing dynamical changes or transitions in pathological states, such as those occurring during the course of clinical treatments or pathogenic processes of disorders. A momentary biomarker is now required for objective monitoring of such dynamical changes. The development of ecological momentary assessment (EMA) allows the assessment of dynamical aspects of diurnal/daily clinical conditions and subjective symptoms. Furthermore, a variety of validation studies on momentary symptoms assessed by EMA using behavioral/physiological/biochemical measures have demonstrated the possibility of evaluating momentary symptoms from such external objective measures. In this review, we introduce physical activity as a candidate biobehavioral biomarker for psychiatric disorders. We also mention its potential as a momentary biomarker for depressive mood. Finally, we address the continuous monitoring of the pathogenic processes and pathological states of depressive disorders based on physical activity, as well as its application in pharmacological animal studies.

## Review

### Seeking a momentary biomarker for psychiatric disorders

Diagnosis of psychiatric disorders largely relies on the description of patients’ subjective symptoms (i.e., physical symptoms and psychological states), causing difficulty in objective evaluation of pathological states of patients. Therefore, there is a recent tendency to incorporate “biomarkers” into psychiatry to improve the current diagnostic system (Singh and Rose 2009). A variety of physiological or biological biomarkers, such as genetic (Lacerda-Pinheiro et al. 2014; Geaghan and Cairns 2015; Banigan et al. 2013), neuroimaging (Wolfers et al. 2015; Arnone et al. 2009; Vita et al. 2009; Wood et al. 2011; Schnack et al. 2014; Diler et al. 2013; Arribas et al. 2010), and biochemical measures (Pagan et al. 2014; Yang et al. 2013), have been identified through association studies with clinical properties of disorders. Some of them have been able to distinguish patients from healthy subjects (Banigan et al. 2013; Arribas et al. 2010; Pagan et al. 2014; Yang et al. 2013) or categorize patients according to their specific psychiatric disorders (Schnack et al. 2014; Nakamura et al. 2007; Diler et al. 2013; Arribas et al. 2010; Banigan et al. 2013) with a certain level of the accuracy. However, they have so far been less successful in capturing dynamical changes in subjective symptoms or transitions of clinical conditions, e.g., those following a clinical treatment (Boksa 2013), at sufficient time-resolutions.

The dynamical or “momentary” aspects of clinical conditions and symptoms are considered important for the diagnosis of the psychiatric disorders and also provide more complete information about the disorders in question. This is widely accepted, especially so in the field of psychosomatic/behavioral medicine (Stone and Shiffman 1994; Conner and Barrett 2012; Trull and Ebner-Priemer 2009; Trull and Ebner-Priemer 2013; Shiffman et al. 2008; Moskowitz and Young 2006), and thus methodological developments to assess momentary information on symptoms, and in the analytical methods for such data (e.g., multilevel modeling), have been implemented over the last two decades.

Among these, ecological momentary assessment (EMA) is a data collection technique capable of repeated real-time assessments of behaviors, psychological states, and physiological reactions in individuals’ daily life (Stone and Shiffman 1994; Kim et al. 2013a). Because of its momentary nature, EMA can enhance the ecological validity of measurements and also avoid the retrospective recall effects which plague in traditional retrospective self-report methods. With the increasing evidence on the usefulness of this technique, EMA is now generally regarded as the ‘gold standard’ to assess the dynamical aspects of subjective symptoms.

Many studies have examined the validity of EMA by investigating covariate properties between momentary self-reported symptoms and other external measures (i.e., biomarkers) using various populations, one of these being patients with psychiatric disorders. For example, cardiovascular reactivity (Kamarck et al. 2005; Kamarck et al. 1998; Smith et al. 2012; Grossman et al. 2008) and cortisol-related reactivity (Robles et al. 2011; van Eck et al. 1996; Smyth et al. 1998; Steptoe et al. 2007; Bitsika et al. 2015) were reported to be associated with levels of psychological stress, and changes in pulmonary functions tested by a spirometer were associated with daily positive/negative affect, as well as the symptom of shortness of breath in asthma patients. Health-related behaviors, such as eating (Lavender et al. 2013; Crosby et al. 2009), smoking (Chandra et al. 2011; Shiffman et al. 2007), and alcohol consumption (Muraven et al. 2005; Jahng et al. 2011), exhibited associations with variation in physical symptoms and psychological states, e.g., craving, positive/negative affect, and anxiety. Furthermore, associations between physical activity measured by self-report and daily fluctuations in psychological states have been reported (Dunton et al. 2009; Wichers et al. 2012). These studies provide strong evidence that various biological/physiological measures are associated with momentary symptoms, possibly in a concurrent fashion. In addition, the existence of such external measures for subjective symptoms indicates the possibility of the practical use of the biomarkers for monitoring momentary symptoms in a continuous fashion simply by measuring other physiological/biological data (i.e., without the need for self-reports).

### Behavioral abnormalities as an objective biomarker

Micro-fluctuations in physical activity contain rich information on the dynamics of our bodily movements in daily life. These data can be continuously obtained in an unobtrusive manner through the use of a wrist-watch-type activity monitor, referred to as an actigraph. The detailed analysis of actigraph data has shown its potential as a psychobehavioral biomarker for psychiatric disorders, and particularly for depressive disorders (Teicher et al. 1995; Teicher et al. 1997; Burton et al. 2013; Indic et al. 2012; Walther et al. 2012; Volkers et al. 2003; Berle et al. 2010). For example, in major depressive disorder (MDD), various types of behavioral alterations were observed; decreased levels of physical activity during daytime (Faurholt-Jepsen et al. 2012; St-Amand et al. 2013; Teicher et al. 1995; Burton et al. 2013); sleep disturbances (Joffe et al. 2009; St-Amand et al. 2013); disruption of the circadian rhythm (Teicher et al. 1997; Robillard et al. 2013; Teicher et al. 1995) as well as improvements over the course of clinical treatment (Baune et al. 2007; Burton et al. 2013; Teicher et al. 1995).

Recent research has shown the existence of robust statistical laws organizing daily life behaviors, specifically how resting and active periods derived from physical activity data are interwoven into daily life (Nakamura et al. 2007). In addition, this research found a significant alteration in the statistical law of resting period durations in patients with MDD, as these patients exhibited more intermittent behavioral patterns than healthy subjects characterized by reduced mean activity levels associated with occasional bursts of physical activity counts (Nakamura et al. 2007; Nakamura et al. 2008). Furthermore, alterations of intermittent properties of physical activity have been reported in schizophrenia and bipolar disorder (Sano et al. 2012; Nakamura et al. 2016). These findings suggest that the intermittency of physical activity is a useful measure for evaluating behavioral abnormalities associated with psychiatric disorders, and that its characterization is likely to provide an objective biomarker for these disorders. Interestingly, it has also been shown that the statistical laws found in human behaviors are shared by mice, and similar alterations in resting period durations to patients with MDD have also been confirmed in mice with mutant circadian clock genes (Nakamura et al. 2008).

### Physical activity provides a momentary biomarker for depressive mood

**Fig. 1:**
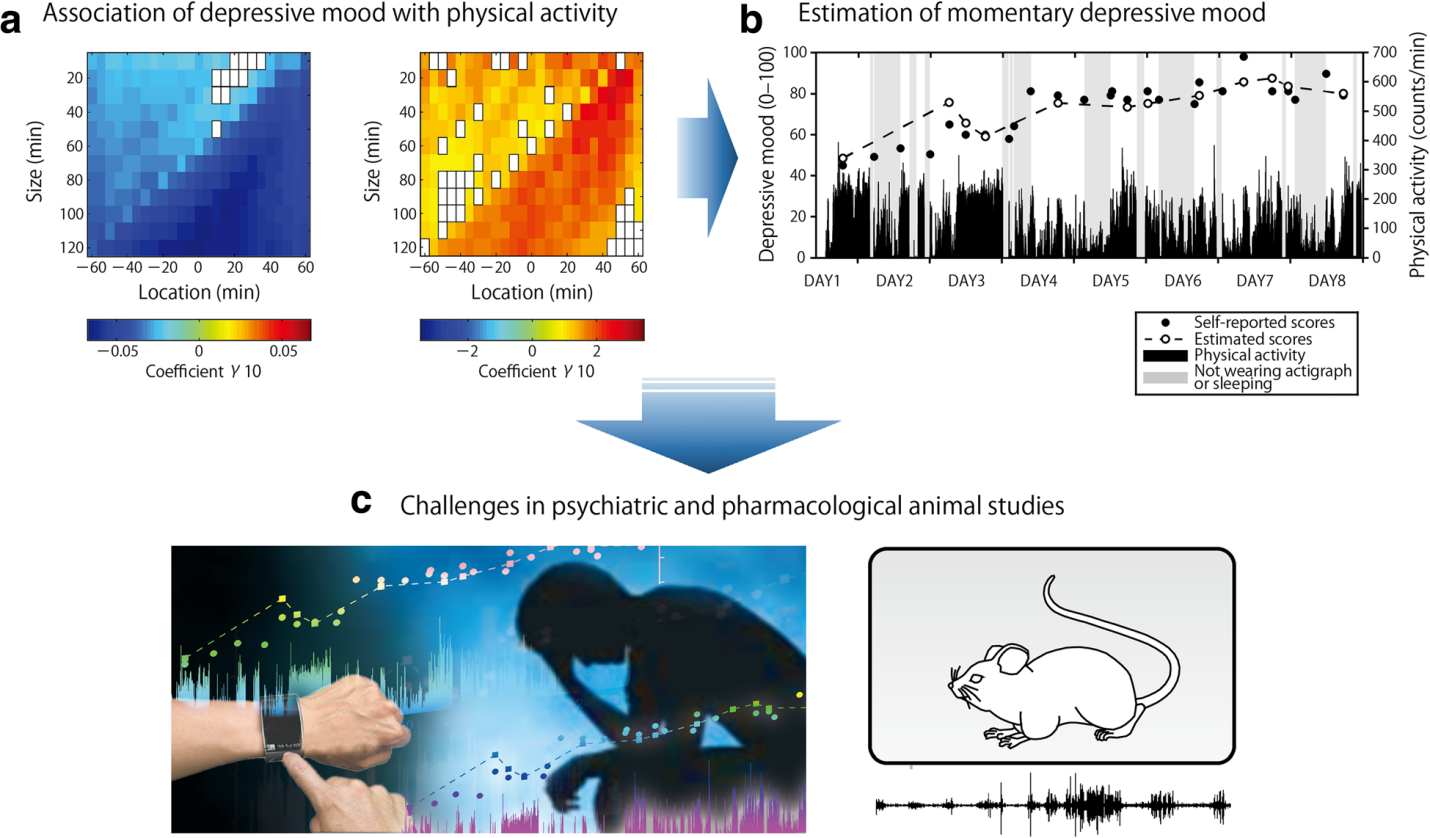
A momentary biomarker for depressive mood. **a** *The temporal associations of depressive mood and local statistics of physical activity*. Estimated values of the univariate multilevel model coefficient for the associations are shown in a colored matrix form consisting of 25 rows (different location) and 12 columns (different size) in patients with MDD (n = 14). Each grid cell indicates specific location and size of a time frame used for calculating the local statistics of physical activity surrounding each EMA recording of depressive mood. A color in each cell represents the value of the model coefficient (γ_10_) of the predictors: local mean (left matrix) or skewness (right one) of physical activity which evaluate lower/higher mean activity levels and asymmetry of a distribution, respectively (i.e., intermittency of physical activity). The false discovery rate with the *q* value of .05 was used as the multiple comparison adjustment. Only the significant cases were shown by colors. Note that the univariate model used for the analysis is as follows; Depressvie mood score_*ij*_ = γ_00_ + γ_10_ (Local statistics of locomotor activity_*ij*_) + ζ_0*i*_ + ε_*ij*_ [see (Kim et al. 2013b) for details]. **b** *A estimation of momentary depressive mood from physical activity in a patient with MDD* [modified from (Kim et al. 2015)]. The parameters of the best-fitting multilevel model describing the temporal associations of depressive mood and local statistics of physical activity were optimized individually using data collected at one week in the early part of the measurement. Subsequently, the momentary depressive scores in another week in the later part of the measurement were estimated using personalized parameters and local statistics of physical activity. In this patient, the correlation coefficient between self-reported (i.e., EMA recordings) and estimated depressive mood scores was considerably high [r = 0.80 (*p* = 0.002)]. Note that the multilevel model we used for estimation is as follows: Depressive mood score_*ij*_ = γ_00_ + γ_10_ (Mean_*ij*_) + γ_20_ (Skewness_*ij*_) + γ_30_ (Mean_*ij*_ × Skewness_*ij*_) + ζ_0*i*_ + ζ_1*i*_ (Mean_*ij*_) + ε_*ij*_. **c** *Challenges in continuous monitoring of depressive disorders and pharmacological animal studies*

More recently, using multilevel modeling approaches (Stone et al. 2007; Schwartz and Stone 1998), Kim et al. probed the psychobehavioral correlates in temporal diurnal fluctuations in momentary depressive mood assessed by EMA and behavioral dynamics (Kim et al. 2013b; Kim et al. 2015). Their results showed that an increased intermittency of physical activity appeared concurrently with the worsening of depressive mood in healthy subjects across a wide range of populations (adolescents, undergraduates, and adult office workers) (Kim et al. 2013b), as well as in patients with MDD (Kim et al. 2015) (Fig. 1-a). Furthermore, the validity of the psychobehavioral correlates across healthy subjects and patients with MDD were confirmed, indicating that the same psychobehavioral correlates are shared by both groups, though only the mean levels of depressive mood scores were significantly higher in the MDD group (Kim et al. 2015).

### Importance of physical activity and further challenges in psychiatric research

Physical activity provides a behavioral biomarker for momentary depressive mood (Kim et al. 2015). Due to the continuous nature of the measurement of physical activity, this behavioral biomarker will enable assessment of diurnal changes of depressive mood with higher resolution than the use of self-reports, contributing to the development of “continuous” monitoring of pathological states in psychiatric disorders (Fig. 1-b and -c). This continuous monitoring has the potential to provide rich information on dynamical aspects of momentary mood and largely contribute to the development of early detection methods of psychiatric disorders (Nakamura et al. 2016) as well as the novel objective evaluation of their treatments.

One further application of the monitoring of symptoms might be in animal research (Fig. 1-c). Animal studies have played a crucial role in psychiatric research (Konopka and Roberts 2015; Cosgrove et al. 2015; Bolkan et al. 2015); however, critical problems exist in the evaluation of animal symptoms, such as “depressive mood.” Diagnosis of humans mainly relies on verbal communication, making it impossible to diagnose animals using the current human diagnostic system. Therefore, animal symptoms are often evaluated based on behavioral assessments which can be impractical to directly apply to humans (Nestler and Hyman 2010). The probing depressive mood based on physical activity may partly solve the above problems and provide a new approach to bridge research about human disorders with animal models.

In order to enhance the accuracy of mood estimation, multidimensional approaches integrating a variety of biomarkers from behavioral, clinical, mathematical, molecular, and imaging studies will be required (Singh and Rose 2009; Kennedy et al. 2012). Due to the complex nature of psychiatric disorders, the presentation of symptoms, process of development, and response to specific medications and treatments for each disease and even each individual are highly diverse. While physical activity is a robust measure, a single biomarker is not likely to be enough to cover this diversity; the combination of physical activity with other behavioral/physiological/biological biomarkers is essential (Kennedy et al. 2012; McGorry et al. 2014). In addition, the development of mathematical methodologies, especially the methods for repeated data with individual variations [e.g., multilevel modeling (Stone et al. 2007; Schwartz and Stone 1998) or machine learning approaches (Bishop 1995)], may become necessary.

One further challenge would be a mathematical modeling approach to psychiatric disorders. The recent development of measurement technologies (e.g., the state-of-the-art wearable devices) has enabled us to obtain high-quality, multidimensional, and intensive longitudinal data (ILD) (Walls and Schafer 2006). This ILD might open possible avenues to reconstruct/infer the dynamical systems underlying the transitions in observed physiological/biological phenomena in psychiatric disorders (e.g., shifts in physical activity data or momentary depressive mood scores). This approach might also provide a novel framework for the early detection of pathological transitions of disease states (Nakamura et al. 2016).

## Conclusion

Physical activity has potential as an objective biobehavioral biomarker for psychiatric disorders. The presence of psychobehavioral correlates between momentary depressive mood and intermittent dynamics of physical activity allow the continuous monitoring of pathogenic processes and pathological states in depressive disorders. It is possible that this approach will also be informative in pharmacological animal studies, and future research should explore these directions.
